# Simulated Microgravity Compromises Mouse Oocyte Maturation by Disrupting Meiotic Spindle Organization and Inducing Cytoplasmic Blebbing

**DOI:** 10.1371/journal.pone.0022214

**Published:** 2011-07-13

**Authors:** Changli Wu, Xinzheng Guo, Fang Wang, Xiaoshuang Li, X. Cindy Tian, Li Li, Zhenfang Wu, Shouquan Zhang

**Affiliations:** 1 Guangdong Provincial Key Lab of Agro-Animal Genomics and Molecular Breeding, College of Animal Science, South China Agricultural University, Guangzhou, Guangdong, People's Republic of China; 2 Department of Animal Science, Center for Regenerative Biology, University of Connecticut, Storrs, Connecticut, United States of America; Texas A&M University, United States of America

## Abstract

In the present study, we discovered that mouse oocyte maturation was inhibited by simulated microgravity via disturbing spindle organization. We cultured mouse oocytes under microgravity condition simulated by NASA's rotary cell culture system, examined the maturation rate and observed the spindle morphology (organization of cytoskeleton) during the mouse oocytes meiotic maturation. While the rate of germinal vesicle breakdown did not differ between 1 g gravity and simulated microgravity, rate of oocyte maturation decreased significantly in simulated microgravity. The rate of maturation was 8.94% in simulated microgravity and was 73.0% in 1 g gravity. The results show that the maturation of mouse oocytes in vitro was inhibited by the simulated microgravity. The spindle morphology observation shows that the microtubules and chromosomes can not form a complete spindle during oocyte meiotic maturation under simulated microgravity. And the disorder of γ-tubulin may partially result in disorganization of microtubules under simulated microgravity. These observations suggest that the meiotic spindle organization is gravity dependent. Although the spindle organization was disrupted by simulated microgravity, the function and organization of microfilaments were not pronouncedly affected by simulated microgravity. And we found that simulated microgravity induced oocytes cytoplasmic blebbing via an unknown mechanism. Transmission electron microscope detection showed that the components of the blebs were identified with the cytoplasm. Collectively, these results indicated that the simulated microgravity inhibits mouse oocyte maturation via disturbing spindle organization and inducing cytoplasmic blebbing.

## Introduction

Microgravity has been reported to induce numerous adverse effects on animals. Studies on astronauts have shown that microgravity is closely associated with bone loss, muscle atrophy, as well as decreased immune [1], [2], [3]. Ideally, real spaceflights should be used to study the effects of space gravity variations, these opportunities, however, are very limited, in addition to the high costs, short duration, deferred reaction, and interference from other conditions aboarding the spacecraft [4]. As a result, simulation models have been designed to test the gravitational effect on the biological systems. Ground-based models have generated reproductive results similar to those obtained from spaceflight, thereby confirming the appropriateness of these models for studying reproductive responses [5]. Recently, it has been reported that simulated microgravity disrupted mouse early embryo development [6]. The effects of decreased gravity on mammalian oocyte maturation, however, have not been investigated.

Mammalian oocyte maturation entails an ordered series of cellular events, including germinal vesicle breakdown, chromosome condensation, spindle formation and migration. The asymmetrical cellular division produces polar bodies and a big, competent, fertilizable oocyte. These events are dependent upon the dynamic changes of oocyte cytoskeleton (microtubules and microfilaments). The cytoskeleton has been described as the structure through which cells sense gravity [7]. Microtubules, which play essential roles during mitosis, including chromosome capture, congregation, and segregation, and cytokinesis [8], [9], were altered and mitosis was prolonged in human breast cancer cell line MCF-7 when cultured under weightless conditions [10]. Microfilaments are disorganized in simulated microgravity through activation of Rho, a serine and threonine kinase [11].

In view of the effects of microgravity on cytoskeleton and the importance of cytoskeleton during oocyte maturation, we hypothesize that the decrease of gravity influences the process of oocyte maturation. The objectives of the present study were to investigate the effect of simulated microgravity on the mouse oocytes maturation *in vitro* and to determine whether the effect is on the microtubules or microfilaments. We used the rotary cell culture system (RCCS) made by National Aeronautics and Space Administration (NASA) to simulate microgravity and studied its effects on mammalian oocyte maturation using the mouse as a model.

## Materials and Methods

### Chemicals and animals

All chemicals were obtained from Sigma Chemical Company unless otherwise stated. Hormones were obtained from Ningbo Hormone Company (Ningbo, China). Female Kunming white mice were obtained from Guangdong Medical and Laboratorial Animal Center (Guangzhou, China).

### Oocyte collection and culture

Animal care and handling were conducted in accordance with policies on the care and use of animals promulgated by the ethical committee of the Guangdong Medical and Laboratorial Animal Center.

Female Kunming White mice at 6–8 weeks of age were injected intraperitoneally with 7.5 IU of PMSG. The females were euthanized 45–48 h post-PMSG administration, and fully grown, germinal vesicle (GV)-intact oocytes were retrieved from each ovary by puncturing the follicles with a 26 gauge sterile needle. The collection medium was M199 with 25 mM HEPES buffer (GIBCO, Cat. No.12350039). The GV oocytes were then transferred into M199 (GIBCO, Cat. No.11150059) supplemented with 10% FCS (Hyclone), 1 IU/ml FSH, 1 IU/ml hCG, 1 µg/ml estradiol, and 0.036 g/l sodium pyruvate, and were cultured in 5% CO_2_ in air at 37°C.

Oocytes were cultured in 50 µl droplets under mineral oil in Falcon petri dishes (Item No.351008) under 1 g gravity. We used the RCCS (Sythecon, USA) developed by NASA to simulate microgravity. The 2-ml high aspect ratio vessels (HARVs) used in the RCCS provide a low-turbulence, low-shear cell culture environment with abundant oxygenation. The rotation randomizes the gravitational vector and leaves cells in a state of constant free-fall, mimicking some aspects of microgravity [12], [13]. After 16 hours culture, the oocytes were collected to examine the maturation rate. The culture experiments were repeated more than 3 times. The total number of oocytes was 1012 in 1 g gravity group, and the total number was 1726 in simulated microgravity group. To detect γ-tubulin, oocytes were collected at 6-hour, 9-hour and 16 hour, respectively. And this experiment was repeated three times.

### Immunohistochemistry and laser-scanning confocal microscopy

The oocytes were fixed at 37°C for at least 30 min in a stabilizing buffer containing 2% formaldehyde, 0.5% Triton X-100, 1 µM taxol, 10 units/ml aprotinin and 50% deuterium oxide. They were then washed three times in a washing buffer (PBS containing 3 mM NaN_3_, 0.01% Triton X-100, 0.2% non-fat dried milk, 2% normal goat serum, 0.1 M glycine, and 2% BSA) and then left in the washing buffer overnight at 4°C for blocking and permeabilization [14]. Oocytes were incubated with mouse momoclonal anti-α-tubulin antibody (Sigma, T9026) at a final dilution of 1∶200 over night at 4°C and then washed three times in the washing buffer. Oocytes were sequentially incubated with fluorescein isothiocyanate (FITC)-conjugated goat anti-mouse secondary antibody (Molecular Probes, F-2761) for 1 h at 37°C to visualize microtubules. To label for microfilaments, the oocytes were subsequently incubated with Rhodamine-Phalloidin (Molecular Probes, R-415) (1∶200) for 30 min at 37°C. Finally, the oocytes were washed, stained for DNA with Hoechst 33342 (Molecular Probes, USA), mounted in PBS containing 50% glycerol, as an anti-fading reagent, and 25 mg/ml NaN_3_ [15], and examined with a Zeiss laser-scanning confocal microscope (LSM510 META DUO SCAN, Germany). To label γ-tubulin, oocytes were incubated with mouse monoclonal anti-γ-tubulin (Sigma, T6557) at a final dilution of 1∶400. The oocytes which stained for γ-tubulin were observed with fluorescent microscope (Nikon).

### Transmission electron microscopy (TEM)

The oocytes were fixed in 2.5% glutaraldehyde in 0.1 M PBS at 4°C, embedded in 1.2% agar. The embedded oocytes were placed in 5% glutaraldehyde to fix for a week at 4°C. Oocytes were washed 6 times with 1% PBS, 20 min each time. They were then fixed for 2 h in 1% OsO_4_ at 4°C and washed 6 times with 1% PBS. After dehydration in graded series of ethanol and acetone, the oocytes were embedded in Epon 812 and ultra-thin sectioned. The sections were stained with uranylacetate and lead citrated for TEM (Tecnai 12, Netherlands).

### Statistical Analysis

All percentages from at least three repeated experiments are expressed as means±SEM. Data were analyzed by independent-sample t-test. *P<*0.05 was considered statistically significant.

## Results

### Simulated microgravity compromises mouse oocyte maturation

**Figure 1:**
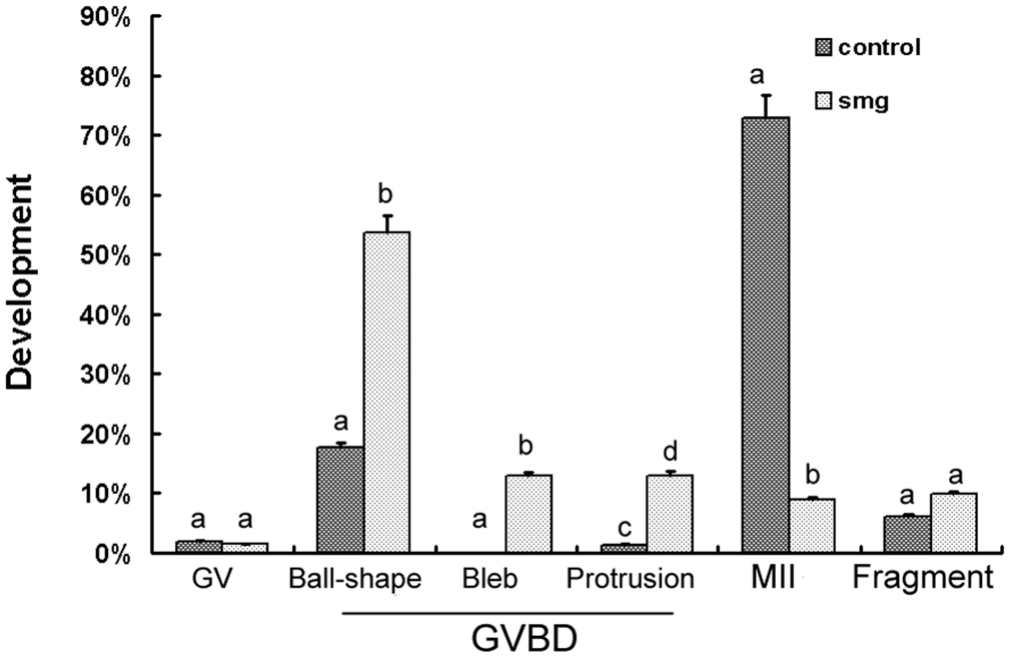
Development of GV oocytes under simulated microgravity. Oocytes were cultured in 1 g gravity (control) and simulated microgravity (smg). After 16 hours cultured, the percentage of different phenotypes were analyzed. This experiment was repeated more than 3 times. Different letters denote statistical differences between groups. a vs b, P<0.01; c vs d, P<0.05.

To investigate the effects of simulated microgravity on mouse oocyte maturation, immature (GV stage) oocytes were cultured for spontaneously maturation under conditions of 1 g gravity and simulated microgravity. 16 hours later, the proportions of GV, GVBD and MII oocytes in the two groups were recorded after the onset of maturation (Fig. 1). At 1 g and simulated microgravity, 1.94%±0.43% (n = 1,012) and 1.52%±0.38% (n = 1,726) oocytes did not undergo GVBD (*P*>0.05), respectively, indicating that the resumption of meiosis was not affected by gravity. On the contrary, extrusion of the first polar body and progression to the MII stage was significantly affected by gravity. While 73.0%±2.6% (n = 1,012) of oocytes extruded the first polar body under 1 g condition, only 8.94%±2.35% oocytes (n = 1,726) did so under simulated microgravity (*P*<0.01). Although oocytes underwent GVBD under simulated microgravity, the oocytes showed different phenotypes observed with light microscope. Most of oocytes still sustained the ball-shape, 12.96% of oocytes underwent cytoplasmic blebbing, and 13.03% of oocytes formed a cytoplasmic protrusion (Fig. 1). Our results indicated that the mouse oocyte maturation was remarkable compromised when cultured under microgravity condition.

### Simulated microgravity disrupts meiotic spindle organization

As mentioned above, 80% oocytes that underwent GVBD could not progress to MII stage. Because the meiotic spindle is the main cellular apparatus responsible for cell division (polar body extrusion), we examined the meiotic spindle under different gravity conditions.

**Figure 2:**
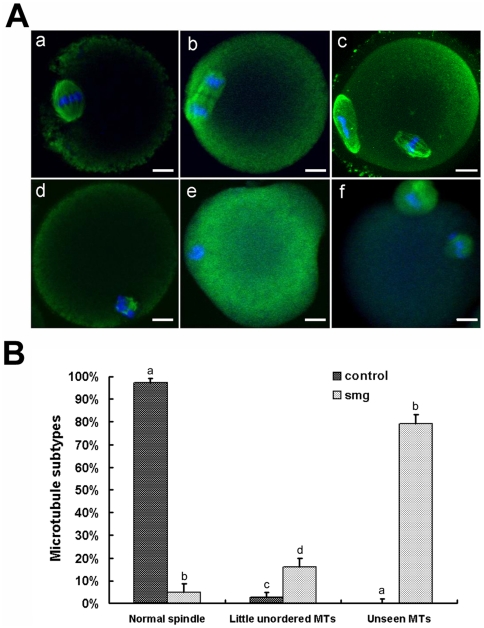
Simulated microgravity disrupts microtubules aggregation in mouse oocytes. A. Spindle morphology of mouse oocytes during meiosis cultured under 1 g gravity (a–c) and simulated microgravity (d–f). a) A mouse oocyte cultured at 6 hours. The barrel-shaped MI spindle is seen with microtubules and chromosomes, arranged on the equatorial plate. b) At 8 hours, chromosomes were separating. c) At 16 hours, the mouse oocyte extruded the first polar body and formed the second spindle. d) At 16 hours, the mouse oocyte showed chromosomes attached with few disordered microtubules. e) At 16 hours, chromosomes without microtubules attachment migrated. f) At 16 hours, the mouse oocyte extruded the first polar body and formed the second spindle. Blue = chromosomes, green = microtubules; Scale bar = 10 µm. B. Percent of microtubules subtypes. Oocytes were cultured in 1 g gravity (control, n = 98) and simulated microgravity (smg, n = 136). After 16 hours cultured, the percentage of different phenotypes were analyzed. Little unordered MTs means that there were little unordered microtubules attached to the chromosomes. Unseen MTs means that there were no obvious microtubules attached to chromosomes. This experiment was repeated 3 times. Different letters denote statistical differences between groups. a vs b, P<0.01; c vs d, P<0.05.

At 1 g gravity, meiotic I spindle was well organized several hours after GVBD, exhibiting a typical barrel shape (Fig. 2A, a). The meiotic II spindle, which is also barrel-shaped, quickly formed after the first polar body extruded (Fig. 2A, c). However, under simulated microgravity condition, typical MI spindle could not form. Chromosomes were able to congregate, but microtubules failed to arrange chromosomes correctly. In some oocytes, condensed chromosomes were surrounded by little unordered microtubules (Fig. 2A,d). And in other oocytes, chromosomes-attached microtubules were not obvious (Fig. 2A, e). In the oocytes which extruded the first polar body under microgravity condition, the meiotic II spindle was also deformed (Fig. 2A, f). As the normal spindle configuration in simulated microgravity was the same with that in 1 g gravity, we did not show the normal spindle picture in simulated microgravity. The percentage of different microtubule subtypes was recorded (Fig. 2B). There were only 4.83%+1.54% oocytes formed normal spindle which were cultured under simulated microgravity. Our results indicated that simulated microgravity disrupted meiotic spindle organization, probably by interrupting microtubules organization, which might be one reason that most oocytes could not extrude the first polar body in microgravity condition.

**Figure 3:**
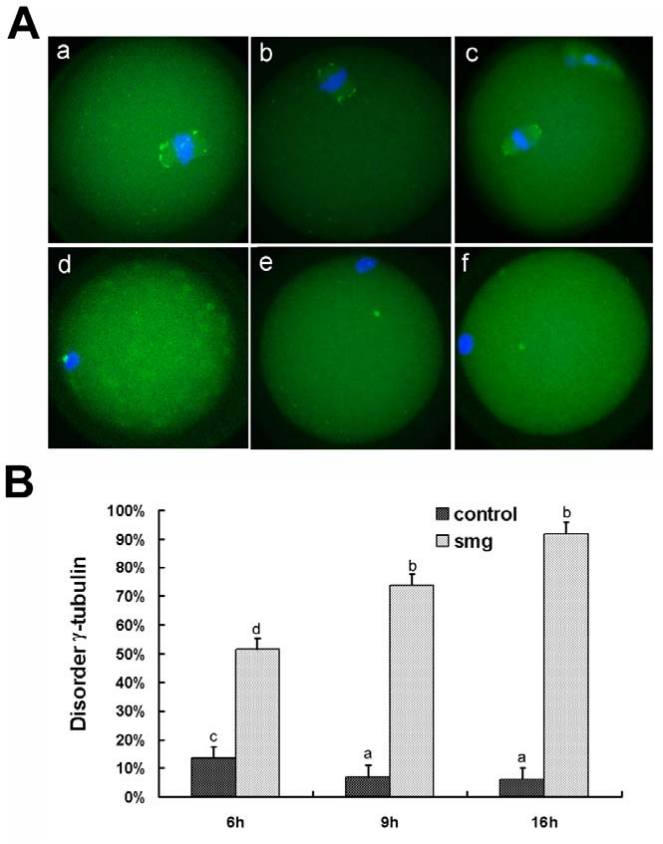
Simulated microgravity disorders γ-tubulin in mouse oocytes. A. Location of γ-tubulin in mouse oocytes during meiosis cultured at different stages under 1 g gravity (a–c) and simulated microgravity (d–f).At 6 (a), 9 (b) and 16 (c) hours, γ-Tubulin accumulated at the spindle poles. d) At 6 hours, γ-Tubulin aggregated around the chromosomes. e) At 9 hours, γ-Tubulin dotted in cytoplasm markedly. f) At 16 hours, γ-Tubulin concentrated into two dots and located in the cytoplasm far away chromosomes. Blue = chromosomes, green = γ-tubulin. Each experiment was replicated 3 times with a minimum of 18 oocytes each group. B. Percent of disorder γ-tubulin configuration in mouse oocytes cultured at different time points under 1 g (control) and simulated microgravity (smg). At 6 h, n = 60(control), n = 91(smg). At 9 h, n = 79(control), n = 128(smg). At 16 h, n = 84(control), n = 117(smg). Every time point experiment was repeated 3 times. Different letters denote statistical differences between groups. a vs b, P<0.01; c vs d, P<0.05.

γ-Tubulin is essential for microtubule nucleation and spindle formation during mouse oocyte meiosis [16], [17]. To determine whether the microtubules disorganized by simulated microgravity was the result of the γ-tubulin disorder, we detected the γ-tubulin and chromosomes. In 1 g gravity, γ-tubulin were located at both poles of the meiotic spindle (Fig. 3A, a–c). Compared to the control, the abnormal γ-tubulin concentrated into dots located around the chromosomes after a 6-hour culture (Fig. 3A, d). At 9 hours, γ-tubulin dotted in cytoplasm markedly (Fig. 3A, e). At the end of the culture, γ-tubulin concentrated into two dots and located in the cytoplasm far away the condensing chromosomes (Fig. 3A, f). The percentage of abnormal γ-tubulin was increased in simulated microgravity along with prolonging culture time (Fig. 3B).

The incidence of abnormal γ-tubulin and the disrupted microtubules suggested that the failure of microtubules organization was the results of the γ-tubulin disorder.

### The function and organization of microfilaments were not disrupted by simulated microgravity

Microfilaments are critical for the migration of meiotic spindle and chromosomes toward the periphery of oocytes [18], [19]. On the other hand, microfilaments are reorganized when meiotic spindle (actually the chromatin) approaching to form a special structure named actin cap, which marks the site of polar body extrusion [20], [21].

**Figure 4:**
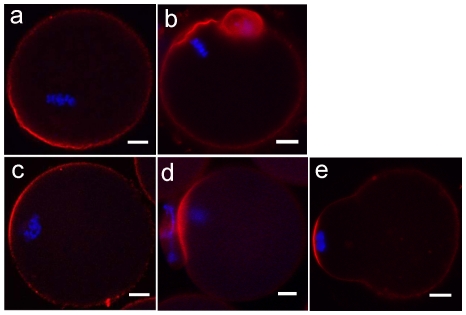
Simulated microgravity does not disorder microfilaments organization and function in mouse oocytes. Immunofluorescence localization of microfilaments (red) in mouse oocytes cultured at 16 hours under 1 g gravity (a, b) and simulated microgravity (c, d, e). a) Microfilaments formed the actins cap, the bright red stain area near the chromosomes (blue). Chromosomes arranged on the plate. b) Oocyte extruded first polar body, microfilaments aggregated in the cortex. c) Microfilaments formed actins cap near the chromosomes. Chromosomes condensed abnormally. d) Oocyte extruded the first polar body. Microfilaments aggregated in the cortex. e) Oocyte formed a protrusion with microfilaments aggregated in the oolemma near the abnormal chromosomes. Scale bar = 10 µm.

In this study, we found the chromatins, whether integrated with microtubules or not, were able to migrate to the oocyte periphery under microgravity condition (Fig. 2A,d and 2A,e), indicating the microfilaments network responsible for spindle or chromatins movement was still functional when gravity was reduced. At the same time, chromatins could also induce microfilaments rearrangement to form actin caps (Fig. 4c, 4d and 4e), showing the organization of microfilaments was not disrupted by simulated microgravity.

Our results show that even though most oocytes failed to mature and microtubules were disorganized in simulated microgravity, the main function and organization of microfilaments was not pronouncedly affected.

### Simulated microgravity induces cytoplasmic blebbing

**Figure 5:**
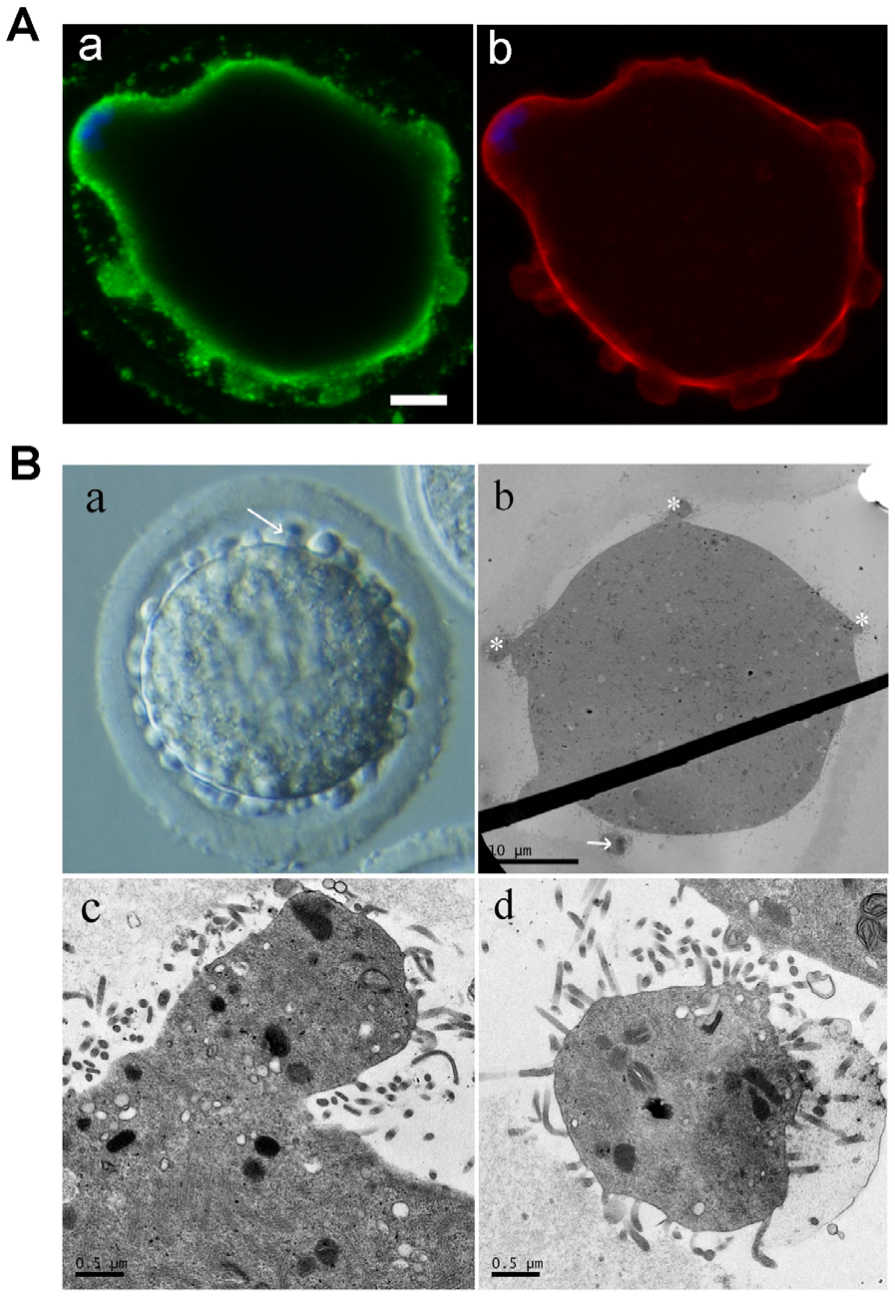
Simulated microgravity induces mouse oocytes cytoplasmic blebbing. Morphology of mouse oocytes with blebs in the perivitelline space cultured in simulated microgravity. A. a) Immunodetection of microtubules (green) and DNA (blue) in blebbing oocytes. b) Immunodetection of microfilaments (red) and DNA (blue) in blebbing oocytes. B. a) An oocyte cultured in simulated microgravity with many cytoplasmic blebs in the perivitelline space (arrow) under normal light microscopy. b) Morphology of oocyte with cytoplasmic blebs in the perivitelline space under transmission electron microscopy. Blebs protruded from the cytoplasm (asterisks) and a free bleb released from the cytoplasm (arrow). c) The ultrastructure of protruded bleb. The blebs do not appear to contain structures other than components of the cytoplasm, and microvilli around the bleb. d) The free bleb in the perivitelline space with microvilli around it. The components of the bleb are identical with the components of the cytoplasm.

Interestingly, in our study, 12.96%±3.24% (n = 1,726) oocytes cultured under microgravity exhibited a distinct but less-defined phenotype, which is marked by many cytoplasmic micro-protrusions around the oocyte (Fig. 5B,a). Under 1 g gravity, however, we rarely observed this phenotype. We also detected the chromatins, microtubules and microfilaments of these oocytes with inmunofluorescence. The concentrated chromosomes were close to the membrane without obvious spindle microtubules (Fig. 5A,a). It also suggests that the microtubules aggregation was disrupted in these blebbing oocytes by simulated microgravity. The microfilaments immunofluorescence showed microfilaments present near the chromosomes and around the blebs in the oolemma and the actin cap was not markedly (Fig. 5A, b). The chromosomes condensation, spindle aberration and cytoplasmic blebbing indicated that failure of meiosis I was because of the failed karyokinesis but not the failed cytokinesis.

To further characterize these blebs, we examined the oocytes by transmission electron microscopy. Blebs are actually projections of the oocyte cytoplasm, and some blebs are detached from the cytoplasm (Fig. 5B, b). No special ultrastructures were observed in the cytoplasmic blebs(Fig. 5B, c–d).

## Discussion

Reproductive functions are changed by microgravity, for example, decreased gravity causes decreases in sperm counts and plasma FSH in males [22], [23], [24] and increases in abortion and changes in maternal behavior [25], [26]. At the cellular and subcellular levels, microgravity changes cell structure, metabolism, behavior and survival [27], [28]. In ground-based studies, the random positioning machine (PRM, a 3D clinostat system), and RCCS are commonly used to simulated microgravity condition. In addition to simulate microgravity, RCCS can be used to generate shear stress to cultured cells [29]. Microgravity condition simulated by RCCS promotes spermatocytes entry into the first meiotic division [30]. In this study, we used the RCCS to simulated microgravity and examined the effects of simulated microgravity on mouse oocytes maturation. Even though microgravity has no effect on the resumption of meiosis or microfilament organization and functions, microgravity profoundly inhibited the polar body extrusion by disrupting the organization of microtubules. As the γ-tubulin is essential for regulating microtubule dynamics and the abnormal distribution of γ-tubulin in simulated microgravity, we speculated that the disorganization of microtubules in simulated microgravity even not all but partially was the results of the disorder of γ-tubulin.

Microtubules are essential component of meiotic spindle, which manages polar body extrusion and distributes genetic materials equally into the polar body and the remaining oocyte. Microtubule nucleation is initiated from γ-tubulin and disrupted γ-tubulin promotes spindle microtubule disorganization [17], [31]. As the oocytes begin to mature, microtubules polymerize around the chromosomes and eventually assemble into the meiotic spindle. Previous studies have showed that microtubule self-organization is gravity-dependent. For human lymphocytes (Jurkat), human epithelial cells (MCF-7), human thyroid carcinoma cells, and swine testicular cells [27], [32], [33], [34], microtubule organizations under simulated microgravity conditions were substantially reduced compared with those under 1 g conditions. In our study, normal meiotic spindles were formed in mouse oocytes under 1 g gravity. But most oocytes cultured in simulated microgravity formed abnormal spindles with few microtubules. Knockdown of γ-tubulin by siRNA in mouse oocytes resulted in smaller spindles with misaligned chromosomes [35]. In this study, the organization of γ-tubulin was disordered in simulated microgravity, which induced the failure of aggregation of microtubules and resulted in abnormal spindle. The results indicate that the microtubule polymerization and organization of mouse oocytes was disrupted by simulated microgravity and resulted in the malformation of intact meiotic spindles. Although the chromosomes migration was not inhibited by simulated microgravity most likely through the actions of microfilaments, the homologous chromosomes can not separate without the function of spindle microtubules. So the oocyte maturation was inhibited by the simulated microgravity through disordered γ-tubulin microtubules organization and disrupting meiotic spindle assembly.

During maturation the oocytes develop cell polarity, which is required for asymmetric cell divisions to extrude polar bodies. Microfilaments are required for chromosomes or spindle migration to the cortex of oocytes and are essential for oocyte polarization. A gravity sensitivity of the actin cytoskeleton has been reported on mouse MC 3T3-E1 osteoblasts [32] and human breast cancer MCF-7 cells [36]. Therefore, we supposed that the oocytes might not polarize themselves under microgravity condition. However, our results showed that the function and organization of microfilaments had not been disrupted by simulated microgravity. Chromosomes could still migrate to oocyte periphery and could induce cortical reorganization of microfilaments to form actin caps as normal gravity. Our results were in accord with the researches on J-111 cells and human SH-SY5Y neuroblastoma, in which cells microfilaments are not affected by microgravity [37], [38]. We reasoned that in different cells types there might be different sensitivities of microfilaments in response to microgravity, or that different cells switch on and off different pathways to react microgravity.

One important finding in our study is that simulated microgravity induces cytoplasmic micro-protrusions of maturing oocytes. 12.96%±3.2% (n = 1,726) oocytes cultured under simulated microgravity were induced cytoplasmic blebbing. This phenotype was rarely observed in 1 g gravity. Until now, available data concerning structural changes during mouse oocyte maturation in simulated microgravity were relatively scarce. Choroidal epithelial cells appear cytoplasmic extensions on apical surface, partial loss of cell polarization in weightlessness and induced alteration in the fine structure of choroids plexus [39]. This was the only one study with cytoplasmic extension in weightlessness we can search. The actin cap was not remarkably in these blebbing oocytes should be because at around anaphase I, oocytes without normal spindle structure can not released from SAC, and microfilaments of actin cap spreaded to the whole oocytes membrane, then induced the cytoplasmic blebbing.

Some animals studies had been conducted on the effect of microgravity on female reproduction, most of these studies focused on the fertilization, embryo development and pregnancy. Fertilization can occur normally under microgravity and preimplantation embryo development might require 1 g environment [6], [15]. Rats kept in space during the latter half of their pregnancies are able to support the growth and development of their gestating offspring [40], [41]. As female astronauts suppress their menstrual cycles during spaceflight [42], relatively little is known about the effect of spaceflight on female reproduction. Whether the oocyte maturation and ovulation are normal during female astronauts in weightlessness is still unclear. Here, our observations suggest that oocytes maturation were compromised under simulated microgravity environment.
